# Comparison of ITS-1 and TBR-1/2 primer sensitivity for the detection of *Trypanosoma evansi* local isolates in experimental rats using a polymerase chain reaction

**DOI:** 10.14202/vetworld.2022.1772-1778

**Published:** 2022-07-24

**Authors:** Endang Suprihati, Lucia Tri Suwanti, Aditya Yudhana, Andika Indra Kusumaningrum

**Affiliations:** 1 Department of Veterinary Science, Division of Veterinary Parasitology, Faculty of Veterinary Medicine, Universitas Airlangga, Surabaya, East Java, Indonesia; 2 Department of Veterinary Medicine, School of Health and Life Sciences, Universitas Airlangga, Banyuwangi, East Java, Indonesia; 3 Department of Veterinary Science, Faculty of Veterinary Medicine, Universitas Airlangga, Surabaya, East Java, Indonesia

**Keywords:** infectious disease, ITS-1, surra, TBR-1/2, tropical disease

## Abstract

**Background and Aim:**

Surra is caused by *Trypanosoma evansi*. The detection method using conventional parasitological tests has not always shown positive results in blood parasite detection, although the livestock has presented with clinical signs. Therefore, a fast and accurate diagnosis is necessary to prevent the disease predominately in field isolates. This study aimed to investigate the sensitivity of molecular detection method using two different specific primers, namely, Internal Transcribed Spacer 1 (ITS-1) and *Trypanosoma brucei* repeat 1/2 (TBR-1/2) against *T. evansi* field isolates from Banten Province, Indonesia.

**Materials and Methods:**

The isolates of *T. evansi* used in this study were collected from Banten Province and cultured and preserved by the National Research Center for Veterinary Science, Indonesia. Eighteen experimental rats were divided into three equal groups, which were categorized as control, 1 × 10^1^, and 1 × 10^4^ infective doses. The isolates were injected into all experimental albino rats intraperitoneally. All samples were tested using conventional blood smear, card agglutination test (CATT), and polymerase chain reaction (PCR) method.

**Results:**

The results of the CATT examination in all treatments showed negative results. However, PCR results showed that two different primers, namely, ITS-1 and TBR-1/2 had been successfully detected *T. evansi* from infected experimental rats, proven by positive PCR band appeared in 480 base pairs (bp) and 164 bp, respectively.

**Conclusion:**

Based on the molecular diagnostic test using PCR method, TBR-1/2 primer is more sensitive to detect *T. evansi* compared to ITS-1 primer. The present finding provides preliminary data for studying the efficiency of different primers if practically applied as a standard diagnostic test for trypanosomiasis, especially in Indonesian livestock.

## Introduction

*Trypanosoma evansi* is a major parasite globally known to cause trypanosomiasis or surra disease in cattle that occur throughout subtropical and tropical regions, including Indonesia [1–3]. *T. evansi* infection is mechanically transmitted to the host by several species of hematophagous flies. Disease manifestations as observed in the field include acute, subacute, and chronic stages, which depend on the virulence of the parasite strain as well as the susceptibility of the host. Genetic variations in *T. evansi* differ in their pathogenicity and biological life cycle, as indicated by parameters such as drug sensitivity and virulence against the host [4, 5]. Various methods have been developed to diagnose surra, including parasitological, serological, and molecular tests [6]. The parasitological test (blood detection in the host) is categorized as the gold standard diagnostic test and is often used in Asian countries such as Iran and India [7, 8]. However, the detection of *T. evansi* using a parasitological test can be used only when there is 60–300 *Trypanosoma*/mL blood [9], which makes it unsuitable for early detection of the disease. Therefore, reaching the number of *Trypanosoma* infection doses will take a long time to be detected. Serological tests can be performed to detect antibody levels in blood serum; however, there is not as specific as there is a high probability of antibody cross-reactivity among other *Trypanosoma* species. Moreover, serological tests cannot differentiate between acute infections and residual antibodies from previous infections [10]. Polymerase chain reaction (PCR) tests can be used to detect the presence of *T. evansi* in blood as they enable rapid and accurate confirmation of results. The success rate of PCR depends on the use of specific primers. However, the sensitivity of PCR is often low when used against field isolates [11].

The prevalence of surra in developing countries such as Indonesia is detrimental to farmers and the government. Therefore, a rapid and accurate diagnosis is essential for early detection and is required to control the disease [12]. In the field, a molecular method, such as PCR using the ITS-1 primer, can detect the presence of *T. evansi*. The ITS-1 primer can be used as the primary standard for detecting the presence of *Trypanosoma* parasites in the host DNA. This is because the ITS-1 primer is detectable in the genus *Trypanosoma* [13, 14]. However, a more specific primer is needed to detect *T. evansi* at the species level to diagnose the disease. The primer TBR-1/2 has been used to detect *T. evansi*. TBR-1/2 has a higher detection sensitivity than other primers used for *T. evansi* [15–17].

Previously, in Indonesia, ITS-1 has been used to detect *T. evansi* from South Sulawesi isolates. Moreover, to the best of our knowledge, there are no reports comparing the sensitivity of PCR to detect *T. evansi* isolates from Banten Province using two different primers, ITS-1 and TBR-1/2. This study aimed to determine the sensitivity of molecular detection of *T. evansi* isolates using two different primers, one specific to the genus and the other specific to the species. The PCR method was also compared with the parasitological (native blood test) and serological (card agglutination test [CATT]) methods, to precisely detect the infection dose of *T. evansi*. In addition to identifying the causative agent of trypanosomiasis, our method can also be applied for early detection and strengthening treatment and prevention measures for trypanosomiasis, especially in Indonesia.

## Materials and Methods

### Ethical approval

The present study was conducted with permission from the National Research Center for Veterinary Science, Ministry of Agriculture, Indonesia. This study was reviewed and approved by the Agricultural Research and Development Committee of the Ministry of Agriculture, Indonesia (certified registration number: Balitbangtan/BBLitvet/Rd/02/2016).

### Study period and location

The study was conducted from April 2016 to October 2016 when trypanosomiasis has categorized as an outbreak in several Provinces in Indonesia. The study was conducted in Banten Province (106.150276 longitude and −6.120000 latitude), approximately 130 – 140 km from Jakarta, the capital city of Indonesia. This area is categorized as endemic for trypanosomiasis, which occurs in livestock. It is considered as the major entrance of imported livestock, especially water buffaloes. Moreover, many water buffaloes were naturally infected with trypanosomiasis due to traditional management and improper sanitation. The samples were processed at Laboratory of Indonesian Research Center for Veterinary Sciences, Ministry of Agriculture, Bogor, Indonesia.

### Experimental animals

The experimental animals used were 2–3-month-old male Sprague-Dawley albino rats, each weighing 80–100 g. The animals were acclimatized at 28°C and 80% humidity for 10 days, and treated with 0.6 mg/kg ivermectin to eliminate possible ectoparasites and endoparasites. During the study period, experimental animals were given commercial pellet feed (Mazuri^®^, Land O’Lakes, Inc., England) and *ad libitum* water. In addition, sterilized husk was used as a base for the cage (litter) to prevent other biological contaminants.

### Trypanosoma samples

The isolates of *T. evansi* used in this study were collected during June - July 2016 from water buffaloes in Banten Province. Then, the isolates of *T. evansi* were preserved using the cryopreservation method in the laboratory of parasitology at National Research Center for Veterinary Science, Ministry of Agriculture, Indonesia. The isolates were injected into 18 experimental albino Sprague-Dawley rats intraperitoneally with 1 × 10^1^ and 1 × 10^4^ infective doses. The study design involved three experimental groups. Each group comprised six rats; the first group was categorized as the control, the second group was injected with 1 × 10^1^ parasite/mL, and the third group was injected with 1 × 10^4^ parasite/mL. The behavioral and clinical symptoms were recorded every day for 10 days; One mL of blood samples for the parasitological test was drawn from the orbital vein of each rat using a hematocrit tube (Sigma-Aldrich, Poole, Dorset, UK). Blood samples were collected in a microcentrifuge tube (Eppendorf, Germany) (volume, 0.5 mL) from day 1 until day 5. A drop of blood was placed on a filter paper (Sigma-Aldrich), which was used for PCR. This was followed by a parasitemia examination using the native scoring method every 2 days until the 8 days. Serological CATT was performed every 4 days. PCR analyses of the blood on the filter paper (Sigma-Aldrich) samples were performed using TBR-1/2 and ITS-1 primers every 4 days until all the infection treatments turned positive. Positive PCR results were obtained on the 3^rd^ day and parasitological examination results on day 8.

### DNA extraction and PCR

Total genomic DNA was extracted from blood samples on filter paper from all experimental groups, namely, control, 1 × 10^1^, and 1 × 10^4^, using the NucleoSpin^®^ Tissue extraction kit (Macherey-Nagel, Germany) following the manufacturer’s protocol. A partial sequence of cytochrome c oxidase 1 (*cox1*) was amplified using the TBR-1/2 forward primer (5′- GAATATTAAACAATGCGCAG-3′) and TBR-1/2 reverse primer (5′-CCATTTATTAGCTTTCTTGC-3′), which were designed from the TBR-1/2 gene of *T. evansi* [15] and the ITS-1 forward primer (5′-CCGGAAGTTCACCGATATTG-3′) and ITS-1 reverse primer (5′-TGCTGCGTTCTTCAACGAA-3′) used to amplify part of the *cox1* region, which were designed from a previous study by Njiru *et al*. [18]. PCR was performed in a 25 μL reaction volume comprising 10 ng template DNA, 2.5 μL 10× FastStart High-Fidelity Reaction buffer (Roche, Mannheim, Germany), 15 mM MgCl_2_, 200 mM dNTPs, 0.2 mM each of forward and reverse primers (Invitrogen, Carlsbad, CA), and 0.625 U FastStart High-Fidelity Enzyme Blend (Roche). The thermocycling conditions (GeneAmp PCR System 9700, Applied Biosystems, Singapore) were as follows: 94°C for 5 min; 35 cycles of 95°C for 30 s, 59°C for 30 s, and 72°C for 45 s; and a final extension step at 72°C for 10 min. For each PCR experiment, a negative, no template control was used along with the other samples. The samples were then separated by 1% agarose gel electrophoresis.

## Results

The results of the CATT examination in all treatments were negative, which indicated that the IgM antibodies were not produced (Table-1). Moreover, it can be seen that the parasitological blood examination method was more sensitive than CATT for the detection of *T. evansi* Banten isolates (Table-1). Even during the initial native blood examination, parasitemia was clearly detected on day 4. However, in the CATT method, no agglutination reaction was detected starting from day 3 until the death of all experimental rats on day 8. The results of parasitemia examination using the native method revealed that the prepatent period with infection doses of 10^1^ and 10^4^ was recorded in the range of 4–8 days and 4–6 days, respectively (Table-1).

**Table 1:** Comparison of *Trypanosoma evansi* detection method using PCR, serological, and parasitological test.

S. No.	Method	Day 1		Day 2		Day 3		Day 4		Day 6		Day 8		Day 9	
						
		10^1^	10^4^	10^1^	10^4^	10^1^	10^4^	10^1^	10^4^	10^1^	10^4^	10^1^	10^4^	10^1^	10^4^
1.	PCR TBR-1/2	+ 33.33% (2/6)	+ 50.00% (3/6)	+ 83.33% (5/6)	+ 83.33% (5/6)	+ 100% (6/6)	+ 100% (6/6)	+ 100% (6/6)	+ 100% (6/6)	+ 100% (6/6)	Death (2/6)	Death (3/6)	Death (6/6)	Death (5/6)	Death (6/6)
	ITS-1	- 0% (0/6)	- 0% (0/6)	- 0% (0/6)	+ 83.33% (5/6)	+ 66.66% (4/6)	+ 100% (6/6)	+ 100% (6/6)	+ 100% (6/6)	+ 100% (6/6)	Death (2/6)	Death (3/6)	Death (6/6)	Death (5/6)	Death (6/6)
2.	Serological (CATT)	- (0/6)	- (0/6)	- (0/6)	- (0/6)	- (0/6)	- (0/6)	- (0/6)	- (0/6)	- (0/6)	Death (2/6)	Death (3/6)	Death (6/6)	Death (5/6)	Death (6/6)
3.	Parasitological	-	-	-	-	-	-	+ 1 50.00% (3/6)	+1 66.66% (4/6)	+2 50.00% (3/6)	+3 33.33% (2/6)	+2 (1/6)	Death (6/6)	+4 16.66% (1/6)	Death (6/6)
		-	-	-	-	-	-	-	+2 33.33% (2/6)	+3 33.33% (2/6)	+4 33.33% (2/6)	+4 33.33% (2/6)	-	Death (5/6)	-
		-	-	-	-	-	-	-	-	-	Death (2/6)	Death (3/6)	-	-	-

PCR=Polymerase chain reaction, CATT=Card agglutination test

**Figure-1:**
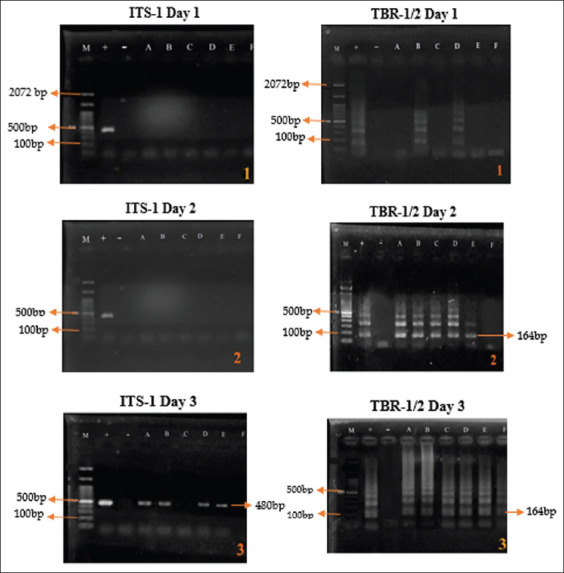
Polymerase chain reaction results of *Trypanosoma evansi* detection using 10^1^ infection dose. M=Maker, +=Positive control, −=Negative control, A-F=Replication.

PCR results showed that both ITS-1 and TBR-1/2 primers successfully identified *T. evansi* from infected experimental rats, with the detection of positive PCR bands at 480 base pairs (bp) and 164 bp, respectively. PCR with ITS-1 primers showed positive results on the 3^rd^ day with the 1 × 10^1^ treatment group. Four out of 6 (66.66%) samples turned positive. PCR with TBR-1/2 primers showed positive bands on the first day of treatment, with 2 out of 6 (33.33%) samples showing positive results (Figure-1).

**Figure-2:**
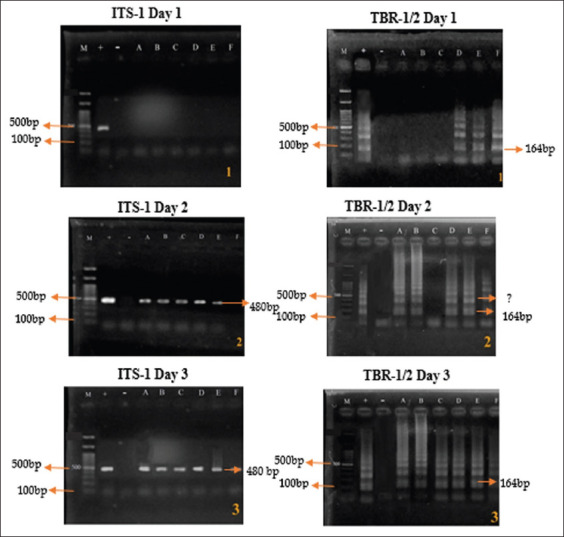
Polymerase chain reaction results of *Trypanosoma evansi* detection using 10^4^ infection dose. M=Maker, +=Positive control, −=Negative control, A-F=Replication.

Moreover, at 1 × 10^4^ infection dose, PCR with ITS-1 showed positive bands in 5 out of 6 (83.33%) since day 2. TBR-1/2, 3 out of 6 (50%) samples were positive from day 1 (Figure-2). Both ITS-1 and TBR-1/2 primers showed no significant differences when used to amplify samples at the highest infection dose of treatment (1 × 10^4^) on day 2 (Figure-2). Similar PCR results were obtained, which revealed 5 (83.33%) positive samples. Based on the PCR results, the ITS-1 primers are effective as standard molecular diagnostic when the infection dose of *T. evansi* is relatively high; ITS-1 primers were not able to detect the parasite when the infection dose of *T. evansi* in the host was still low sensitive compared to that with the TBR-1/2 primers (Figure-2).

## Discussion

The present study revealed not only the sensitivity of molecular identification using PCR with ITS-1 and TBR-1/2 primers but also its accuracy in detecting *T. evansi* local isolates when compared to the blood smear and CATT methods. Parasitemia is difficult to detect at an early stage as the clear morphology of the blood parasites cannot be identified with conventional parasitological tests with a low-level phase makes it more difficult to observe. For example, a Giemsa-stained blood smear does not always indicate the presence of *Trypanosoma*, although the animal host presents clinical signs of the disease [16, 17]. Moreover, microscopic examination of blood smears indicates that it is less sensitive and efficient for it to be widely used for screening trypanosomiasis in field cases [18]. However, the blood smear method is still the most commonly used method for diagnosing trypanosomiasis. In the present study, the parasitological method showed positive results on day 4 of infection, with only two positive results out of six blood samples obtained using the Giemsa staining method (Table-1). The present results are also in accordance with a previous study conducted by Ahmadi *et al*. [19], which detected four positive results out of 117 blood samples. Zangooie *et al*. [14] reported zero positive results with microscopic examination of 113 blood samples, whereas seven samples were positive with a PCR method. A similar study in Indonesia [3] found only one positive sample out of 100 blood samples using the blood smear method.

In the results with PCR method, the TBR-1/2 primer was found to be more sensitive than ITS-1 primer in detecting *Trypanosoma* spp. infection (Figure-2). The present study is supported by Fernández *et al*. [20] that the TBR-1/2 primer is suitable for diagnosis, where infection dose of 1 × 10^2^ parasites/mL blood was detectable at 12 h after infection; PCR with ITS-1 primer showed positive results. This is because the TBR-1/2 primer can amplify *T. evansi* DNA at 0.001 ng, whereas the ITS-1 primer requires *T. evansi* DNA at a concentration of 0.01 ng. These numbers were arrived at by performing a PCR with different concentrations of pure *T. evansi* DNA with each of the primers, which can determine the sensitivity of the primers. With respect to the target sequences in *T. evansi* genome, TBR-1/2 primer targets 10,000–20,000 multicopy satellite regions, while ITS-1 primer targets 100–200 satellite regions; TBR-1/2 can, therefore, be used as the gold standard for the detection of *T. evansi* infections, especially in field isolates [20, 21]. Moreover, Nakamura *et al*. [22], also reported that ITS-1 combined with serum resistance-associated primers is suitable for detecting *Trypanosoma* spp. in cattle hosts and fly vectors. However, the combination of ITS-1 and TBR-1/2 primers in detecting *T. evansi* has been applied for the 1^st^ time in this study. In accordance with a previous study in Egypt, the TBR-1/2 primer is more sensitive in detecting *T. evansi* than the rode Trypanozoon antigen type (RoTat) primer, which is less sensitive [23].

The size of DNA fragments amplified with the ITS-1 primer was 480 bp. ITS-1 primer can identify several species of *Trypanosoma* parasite because it has an isoform gene for a detectable species. The length of ITS-1 PCR products in several species of trypanosomes was 700 bp for *Trypanosoma congolense savannah*, 400 bp for *Trypanosoma simiae*, 250 bp for *Trypanosoma Vivax*, and 480 bp for *T. evansi*. The ITS-1 DNA fragment length for detecting *T. evansi* is similar to the DNA fragment length of *T. brucei subspecies* [12, 21]. ITS-1 primer has been widely used for molecular epidemiological studies, especially in African trypanosomiasis. Moreover, the ITS-1 primer has an advantage over other primers in that it can generate different PCR products when used in a sample containing two or more *Trypanosoma* species that frequently infect livestock [11]. Njiru *et al*. [18] also mentioned that the ITS-1 primer could identify *Trypanosoma* species simultaneously in infected samples with more than 1 type of *Trypanosoma*. In South Africa, the ITS-1 primer could also identify multiple infections where several species of *Trypanosoma* infected water buffaloes and cattle; this aspect of ITS-1 primer proves advantageous in that local farmers can reduce diagnostic costs and this provides faster results compared to single primers for one type of *Trypanosoma* [18, 23].

In the present study, amplification with the TBR-1/2 primer produced more than 1 DNA band; the presence of extra DNA bands with this primer varied depending on the sample being tested. However, in the blood sample, there was a DNA fragment located at 164 bp, indicating that the sample was positive for *T. evansi* infection. A similar finding was reported in a previous study conducted by Ramírez-Iglesias *et al*. [5] concluded that tandem repeats were present in the DNA target. Furthermore, the occurrence of multiple DNA bands with the TBR-1/2 primer was presumably due to amplification as a result of primer attachment to the repeated regions of *Trypanosoma* DNA; this situation can also be triggered by a large number of DNA templates that are abundant [23]. The TBR-1/2 primer in the previous study was not only used to identify *T. evansi* but was also capable of detecting *T. brucei* coding gene with a band length shorter than 164 bp [24]. Moreover, the TBR-1/2 primer also has the advantage of not causing cross-reaction with *T. vivax* and is more sensitive if used to detect *T. evansi* in the infected host [5]. The differences between our results and those obtained in the previous study conducted by Fernández *et al*. [20], may be due to different primer sets and DNA extraction methods, diversity of trypanosome strains, and different PCR conditions. In this study, Chelex resin was used for DNA extraction from the blood samples. The method provided a high DNA yield with suitable purity, was less complicated, and overcame the toxic effects and risk of using organic extraction methods [25].

Surra is a serious infectious disease with various prevalence rates with significant morbidity and mortality among livestock in Africa, South America, and Asia, including Indonesia [26]. In the Middle East, such as Palestine, the overall trypanosomiasis prevalence of 18% was similar to the infection rate of neighboring countries in the region. Moreover, a previous study in Saudi Arabia reported that the infection rates were lower in donkeys and horses, with a total prevalence of 3.3% and 2.8%, respectively [27]. Different results from Egypt have reported that no infection was detected in donkeys and horses; however, surra disease was recorded in camels, with a 31.4% prevalence rate [28]. In South Sulawesi, Indonesia, the prevalence rate of surra was 0% when detected using the conventional parasitological method; however, a 3% prevalence rate was detected in cattle using PCR as a molecular diagnostic method [3]. Therefore, a PCR method using more sensitive primers is required for early diagnostic testing. To the best of our knowledge, this is the first study to briefly compare the sensitivity of ITS-1 and TBR-1/2 primers when used for molecular diagnostics using PCR. Our results also confirm that molecular diagnostics using TBR-1/2 primers are more efficient if applied as a gold standard to identify *T. evansi*, especially in field isolates, because the TBR-1/2 primer is not only proven as specific but also as a sensitive primer compared to ITS-1.

## Conclusion

Based on the molecular diagnostic test in blood samples from experimentally infected rats using PCR, the TBR-1/2 primer was more sensitive to detect *T. evansi* local isolate than the ITS-1 primer. Notably, conventional parasitological tests can also identify the microscopic morphology of *T. evansi* if parasitemia has occurred in the host during the peak phase of infection. The present findings provide preliminary data for studying the sensitivity and efficiency of different primers if practically applied as a standard diagnostic test for trypanosomiasis, especially in Indonesian livestock. However, the present method only applied one species of *Trypanosoma* parasite and it remains to be seen unknown if more than 1 species of *Trypanosoma* can be detected. Furthermore, the present study provides beneficial data for future studies using different species of *Trypanosoma* and recommendations for parasitic disease prevention measures using the molecular approach as a standard early diagnostic method.

## Authors’ Contributions

ES: Supervised the study and sample collection. AIK: Carried out sample collection and performed the standard parasitological examination. LTS: Carried out molecular identification and data interpretation. AY: Experimental materials collection and data analysis. All authors contributed to the drafting and revision of the manuscript. All authors have read and approved the final manuscript.
